# Supplementary material for: Dual role of USP30 in controlling basal pexophagy and mitophagy
Source: EMBO Rep. 2018 Jun 12;19(7):e45595. doi: 10.15252/embr.201745595 (PMC6030704; doi:10.15252/embr.201745595)

Appendix

Table of Contents

Figure S1: Analysis of PEX5 in USP30 WT and KO cells.

Figure S2: Analysis of protein ubiquitylation in USP30 WT and KO cell lysates and mitochondria enriched fractions.

Figure Legend Appendix

Appendix Figure S1 –PEX5 is not differentially ubiquitylated in USP30 KO lysates.
(A) Validation of PEX5 antibody in U2OS and hTERT-RPE1 cells transfected for 72 h with non-targeting (NT1), PEX5 and PMP70 targeting siRNA.
(B) Representative western blot of hTERT-RPE1 WT and KO cells (WT1, WT3, KO2 and KO6) analysed under reducing (+DTT) and non-reducing (-DTT) conditions to assess Cys11-ubiquitylation of PEX5.
(C) Representative western blot of hTERT-RPE1 WT and KO cells (WT3 and KO2) treated for 6 h with 0.4 mM H_2O_2 and/or 100nM Epoxomycin (Epox), lysed in buffer containing 20 mM Tris-HCl pH 7.5, 150 mM NaCl, 1 mM EGTA, 1 mM EDTA, 1% Triton X-100 and protease inhibitors. Black arrow-head indicates full length, red and blue arrowhead, modified PEX5 species.
(D) Representative western blot of endogenous PEX5 immunoprecipitated from hTERT-RPE1 WT and KO cells (WT3 and KO2) treated for 6 h with 0.4 mM H_2O_2 or 100 nM Epoxomycin (Epox) and lysed in 20 mM Tris-HCl pH 7.5, 150 mM NaCl, 1 mM EGTA, 1 mM EDTA, 1% Triton X-100 and protease inhibitors. Black arrow-head indicates full length, red and blue arrowhead, modified PEX5 species.
(E) Representative western blot of 20K membrane pellets (incl. peroxisomes) derived from hTERT-RPE1 WT and KO cells (WT1, WT3, KO2 and KO6). Cells were homogenised in HIM buffer supplemented with fresh proteases inhibitor alone or with 10 mM NEM. A post-nuclear supernatant (PNS) was obtained and first centrifuged for 15 min at 7000 g. The post-mitochondrial supernatant was then centrifuged at 20,000 g for 15 min and the pellet (peroxisomes enriched fraction) was resuspended in SDS-sample buffer and subjected to western blot analysis.

Appendix Figure S2 – Ubiquitylation profile of USP30 WT and KO cell lysates and mitochondria enriched fractions.
(A) Representative western blot of a TUBE (Life Sensor, UM402) pull down from hTERT-RPE1 WT and KO cells (WT3 and KO2), treated for 6 hours with 100 nM Epoxomycin, then lysed in 50 mM Tris-HCl pH 7.5, 150 mM NaCl, 1 mM EDTA, 1% NP40, 10% Glycerol and fresh protease inhibitors. Equal amount of lysates were incubated with agarose-TUBE beads for 1 h at 4°C. After three washes in TBS including 0.1% Tween, samples were eluted in SDS-sample buffer, incubated 5 min at RT and analysed by western blotting. Membranes were incubated overnight either just with VU1 antibody (total ubiquitin) or first incubated with GFP-K6 affimer (Avacta, 0.1 µg/ml– [1]) prior to incubation with anti-GFP.
(B) Representative western blot of a TUBE pull down from hTERT-RPE1 WT and KO cells (WT1 and KO2), treated for 2 h with Oligomycin A and Antimycin A (both 1 µM, O/A), then lysed as in (A). Both input (15 µg) and beads were analysed by SDS-PAGE and immunoblotted with TOMM20 antibody.
(C) Representative western blot of purified mitochondria from hTERT-RPE1 WT and KO cells (WT1 and KO2). Mitochondria were isolated using the commercially available Mitochondria Isolation Kit (Miltenyi Biotec, 130-094-532) following the manufactures' instructions with minor modifications.

1. Michel MA, Swatek KN, Hospenthal MK, Komander D (2017) Ubiquitin Linkage-Specific Affimers Reveal Insights into K6-Linked Ubiquitin Signaling. *Mol Cell* **68:** 233-246 e235

Appendix Figure S1

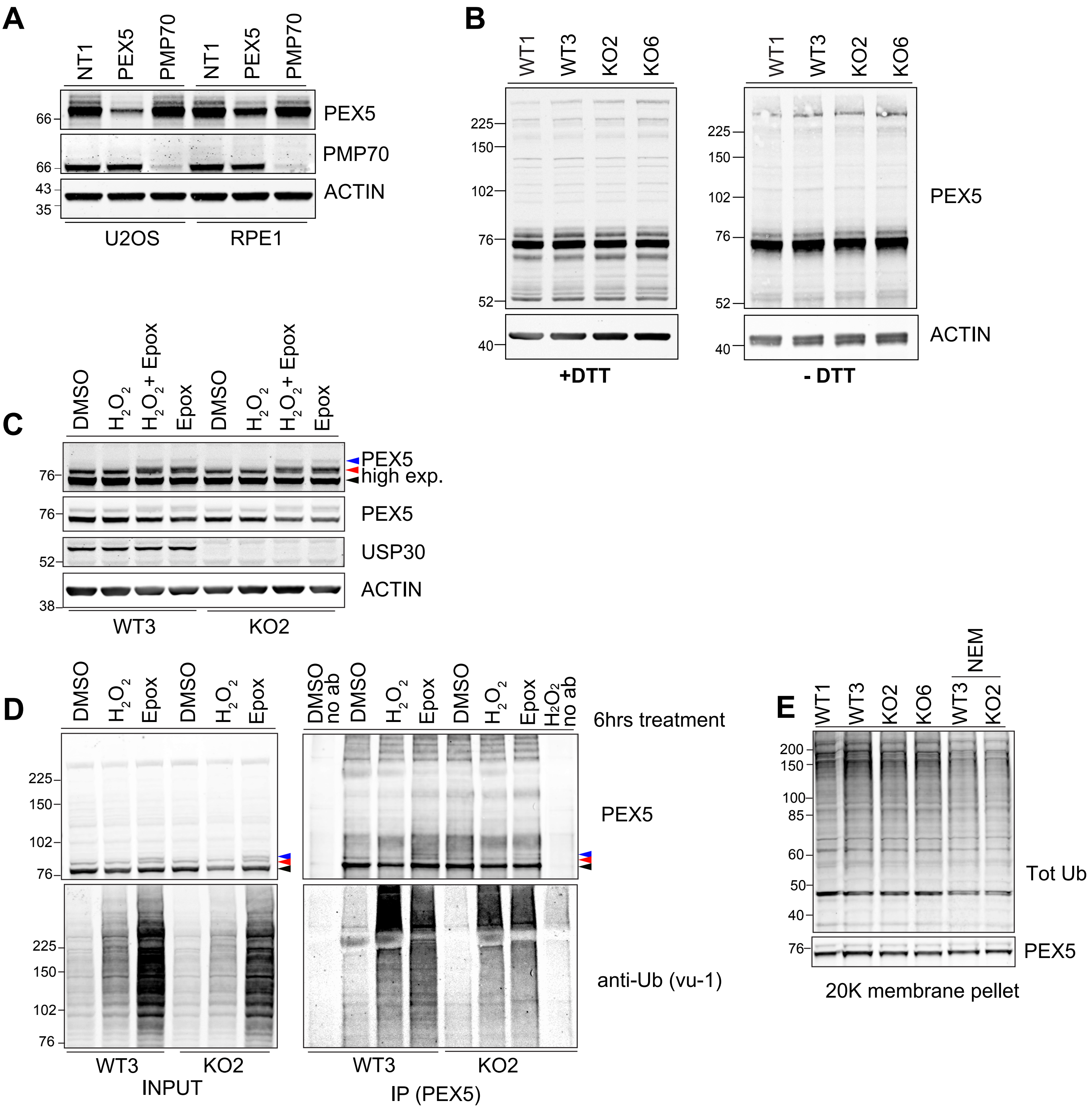

Appendix Figure S2

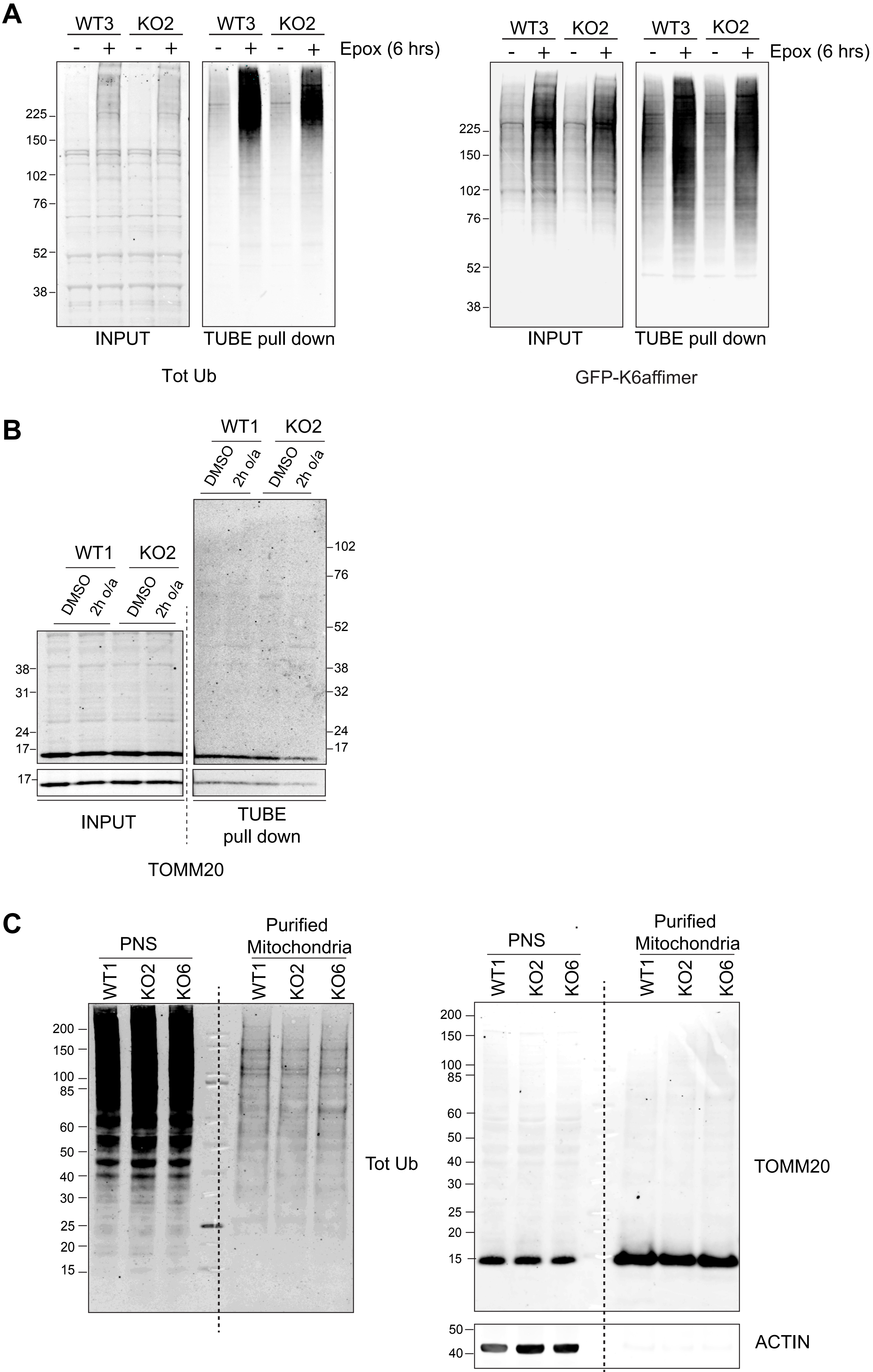

Supplement: Supplementary file 1 — Appendix [file EMBR-19-e45595-s001.pdf]
