# Supplementary material for: Dual role of USP30 in controlling basal pexophagy and mitophagy
Source: EMBO Rep. 2018 Jun 12;19(7):e45595. doi: 10.15252/embr.201745595 (PMC6030704; doi:10.15252/embr.201745595)
Supplement: Supplementary file 2 — Expanded View Figures PDF [file EMBR-19-e45595-s002.pdf]

Expanded View Figures

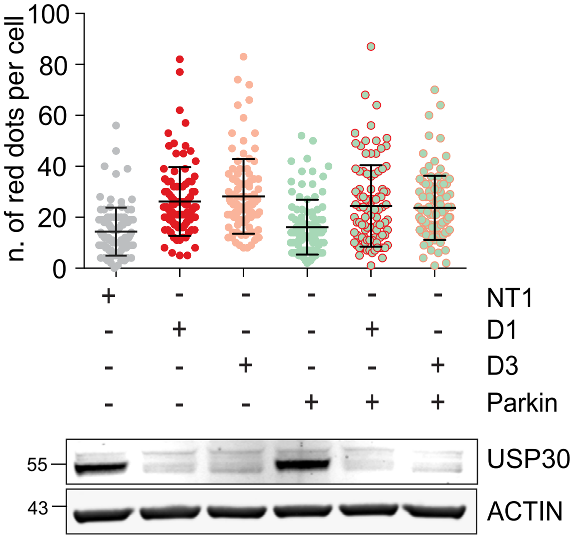

Figure EV1. USP30 regulates basal mitophagy independently of Parkin.

U2OS-MGFIS cells were treated with either non-targeting siRNA (NT1) or with siRNA targeting USP30 (D1, D3) or Parkin, and analysed by 3i-spinning disk confocal microscopy. The number of red mCherry-GFP-Fis1$_{(101-152)}$-positive puncta (dots) per cell was quantified in three independent experiments (average ± SD, 20 cells per experiment). Also shown is a representative Western blot.

Source data are available online for this figure.

© 2018 The Authors

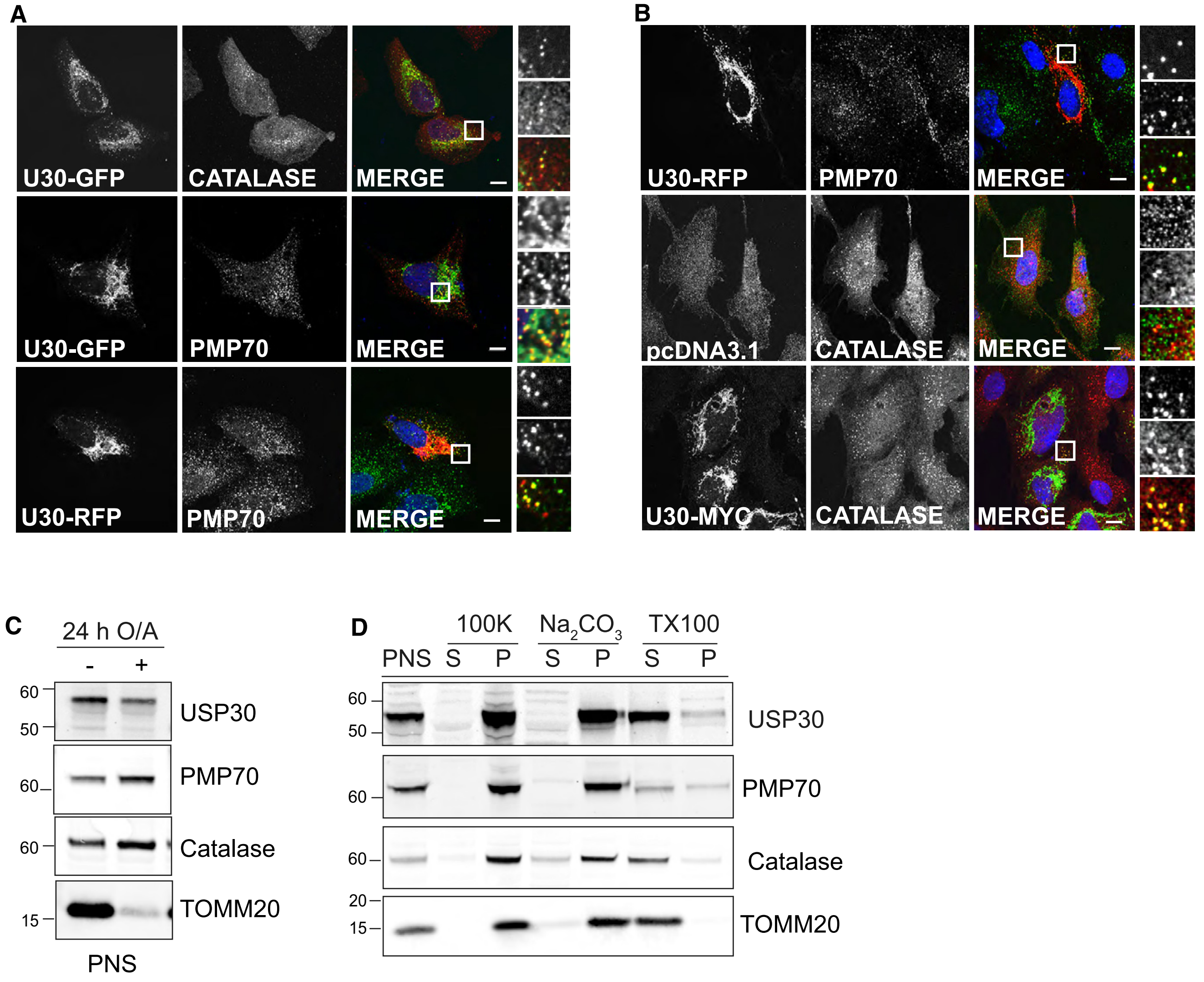

Figure EV2. GFP-, RFP- and Myc-tagged USP30 co-localises with the peroxisomal proteins catalase and PMP70 in U2OS and hTERT-RPE1 cells.

A U2OS cells were transfected with pEGFP-N3-USP30 or pRFP-N3-USP30 for 24 h, fixed and stained for the peroxisomal proteins, catalase and PMP70.

B hTERT-RPE1 cells were transfected with pRFP-N3-USP30, pCMV6-myc-DDK-mUSP30 (Origene) or pCDNA-3.1 for 24 h, fixed and stained for Myc or the peroxisomal proteins PMP70 and catalase. Scale bars (A, B) 10 μm.

C hTERT-RPE1 YFP-Parkin cells were either left untreated or treated for 24 h with oligomycin A (1 μM) and antimycin A (1 μM). A post-nuclear supernatant (PNS) was obtained and analysed by SDS–PAGE to assess mitochondrial and peroxisomal protein levels. Representative experiment ($n = 2$).

D A membrane pellet obtained from untreated hTERT-RPE1 YFP-Parkin cells was incubated with 0.1 M alkaline Na_2CO_3 or 2% Triton X-100 and 1 M NaCl, then subjected to ultracentrifugation. Supernatants and pellets were analysed by SDS–PAGE. Representative experiment ($n = 2$).

Source data are available online for this figure.

© 2018 The Authors

Figure EV3. Basal autophagic flux and peroxisome abundance are not affected in USP30 KO cells.

A Representative images of hTERT-RPE1 cells transfected with Keima-SKL and CFP-LAMP1 for 48 h, fixed and analysed by 3i-spinning disk confocal microscopy. Scale bar: 10 μm. Arrows indicate Keima-SKL "red" puncta that colocalise with the lysosomal marker CFP-LAMP1.

B Representative Western blot of cells analysed in Fig 5B.

C Keima-SKL "red" puncta in USP30 KO or WT hTERT-RPE1 cells. Keima-SKL was either transfected on its own or together with USP30-GFP and USP30C77S-GFP for 48 h. Two independent experiments were analysed, 20 cells per experiment, mean ± range.

D Quantification of the percentage of mt-Keima "red" puncta in USP30 KO6 or WT1 hTERT-RPE1 cells (n = 2 independent experiments, 20 cells per experiment, mean ± range).

E hTERT-RPE1 USP30 WT and KO cells (WT1, WT3, KO2 and KO6) were transfected with RFP-GFP-LC3b for 48 h prior to imaging by 3i-spinning disk confocal microscopy. Shown are representative images and the numbers of RFP-GFP-LC3b puncta per cell as an assessment of basal autophagic flux. Two independent experiments were analysed, 20 cells per experiment, mean ± range.

F hTERT-RPE1 cells were transfected with non-targeting (NT1) or USP30 targeting siRNA (D1 and D3) for 72 h and lysed in RIPA buffer. Protein samples were analysed by SDS–PAGE and Western blots probed for catalase, PMP70, PEX5 and PEX19.

G hTERT-RPE1 USP30 WT and KO cells (WT1, WT3, KO2 and KO6) were lysed in RIPA buffer and analysed by SDS–PAGE and Western blots probed for catalase, PMP70, PEX5 and PEX19.

H Quantification of the number of peroxisomes of hTERT-RPE1 USP30 WT and KO cells (WT1 and KO2), fixed and stained for PMP70 (AlexaFluor488) and catalase (AlexaFluor594). Graph shows the data from three independent experiments in which 10 cells per experiment were analysed.

I Colocalisation analysis of hTERT-RPE1 USP30 WT and KO cells, fixed and stained for the peroxisomal protein PMP70 (AlexaFluor488) and catalase (AlexaFluor594). Graphs show Manders' coefficients between catalase and PMP70 or PMP70 and catalase derived from z-stacks of 10 cells per experiment (n = 3 independent experiments, 10 cells per experiment). Note that PMP70 is a component of both immature and mature peroxisomes whereas catalase is associated only with import-competent peroxisomes. Scale bars 10 μm.

Source data are available online for this figure.

© 2018 The Authors

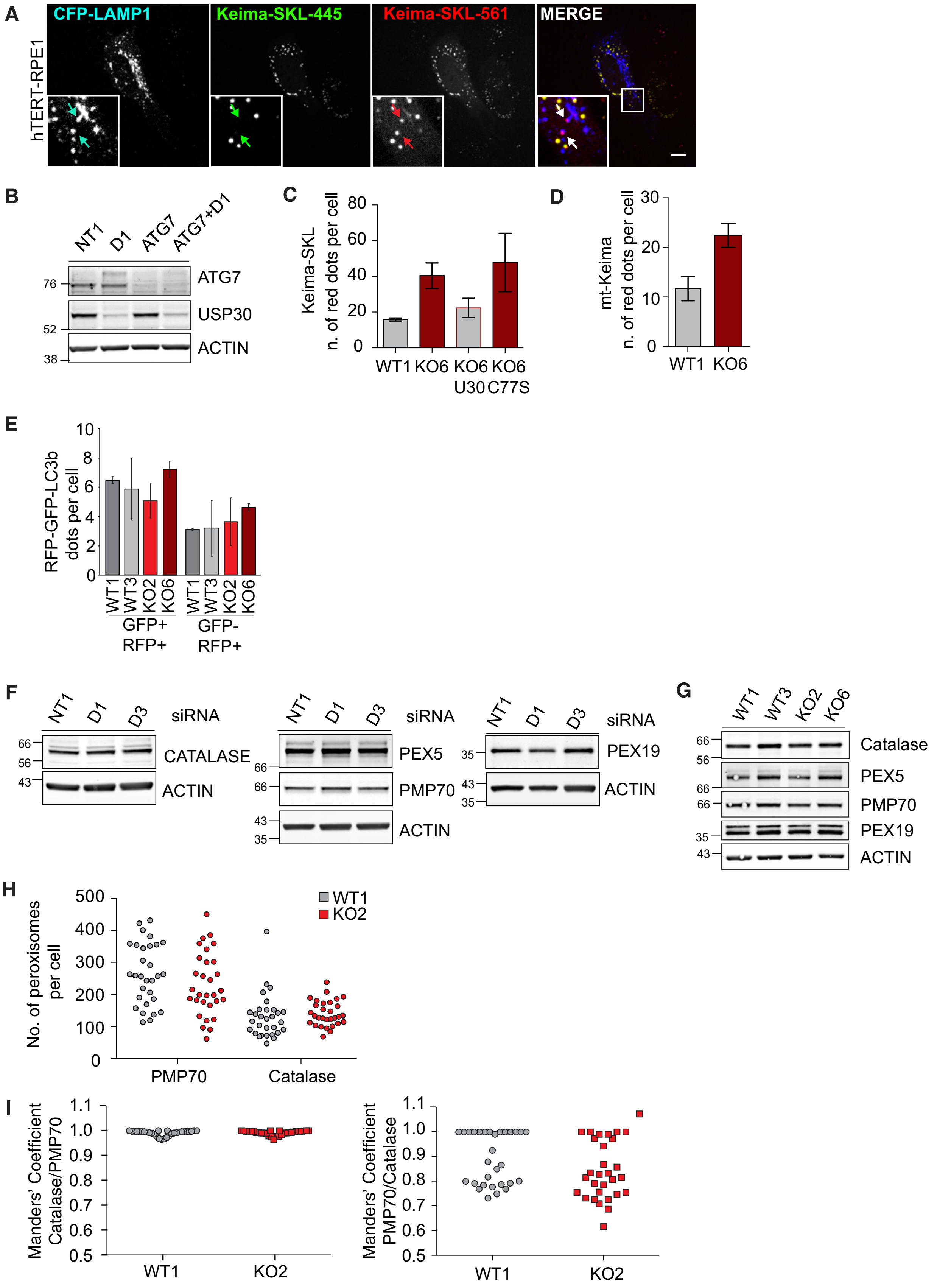

Figure EV3.

© 2018 The Authors
