# Supplementary figures and images for: Dual role of USP30 in controlling basal pexophagy and mitophagy
Source: EMBO Rep. 2018 Jun 12;19(7):e45595. doi: 10.15252/embr.201745595 (PMC6030704; doi:10.15252/embr.201745595)

FigEV1A

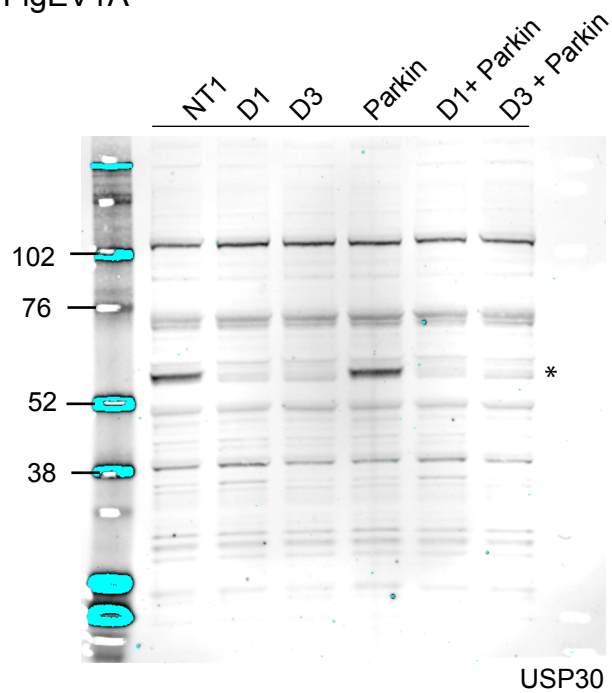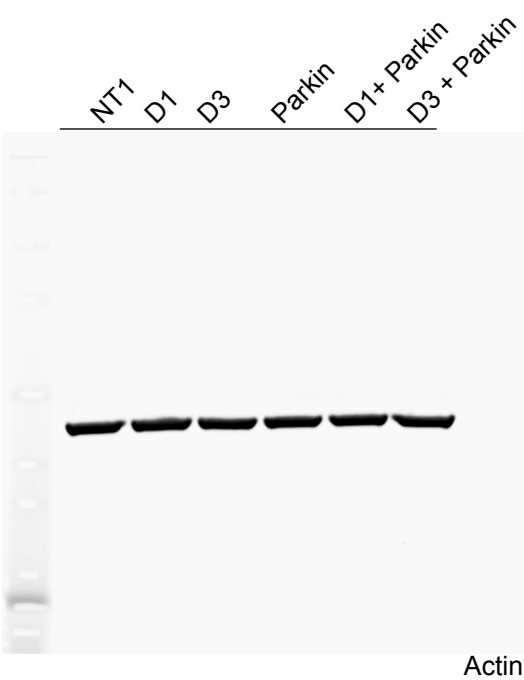

EV2C

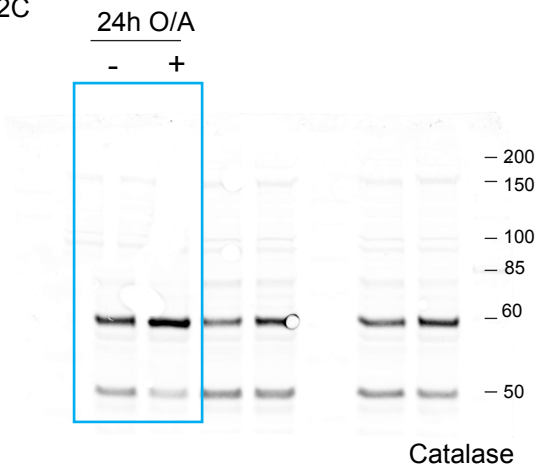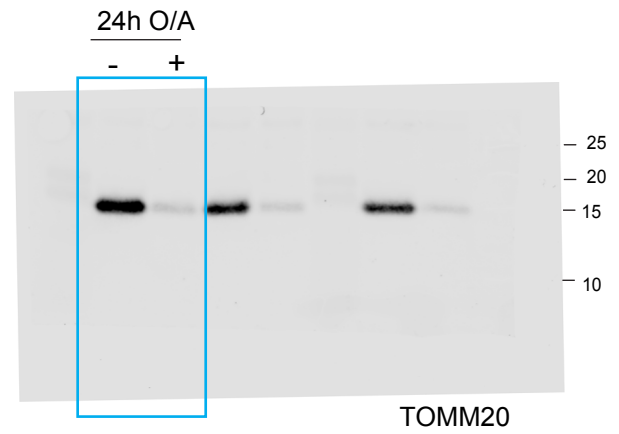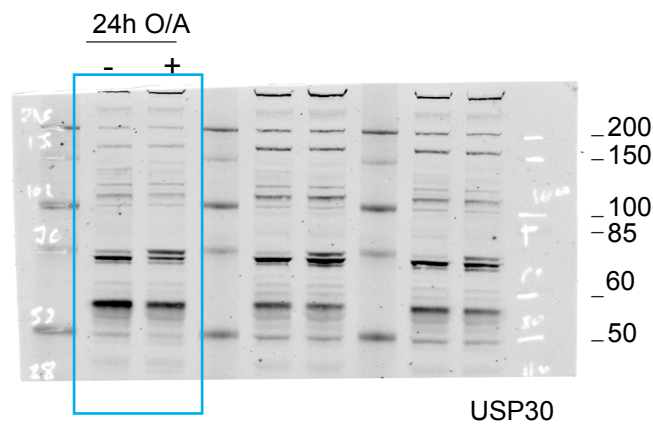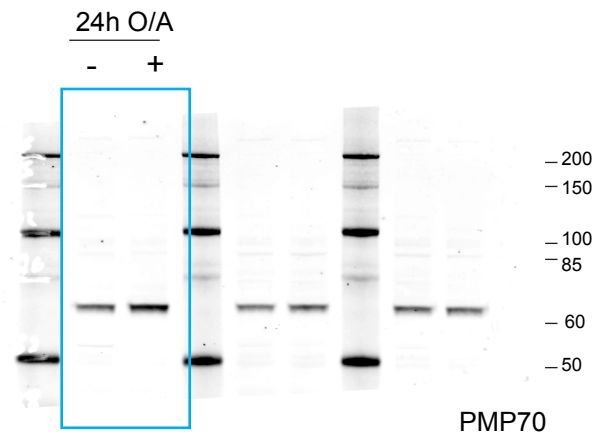

EV2D

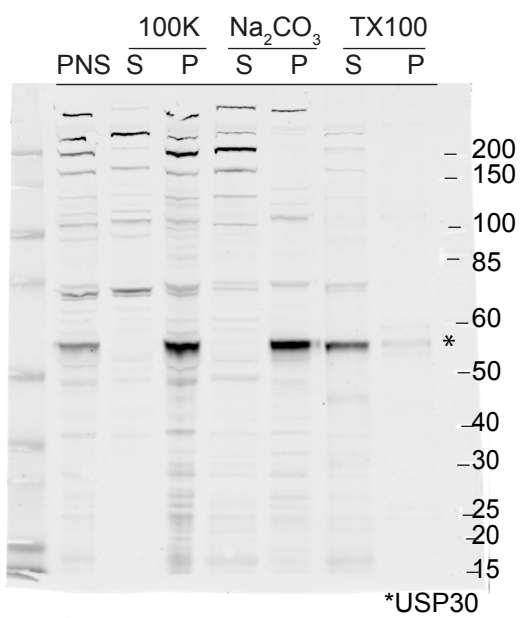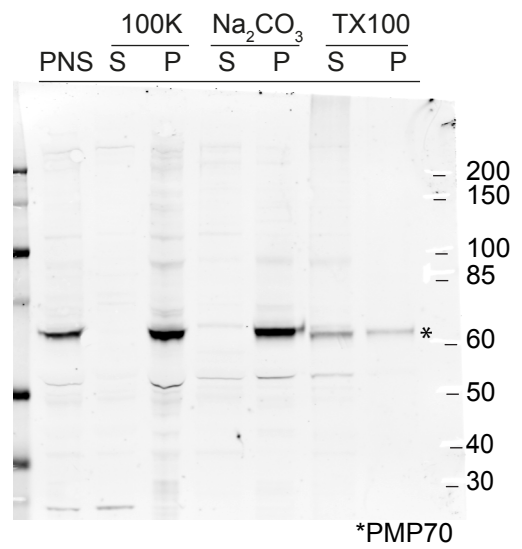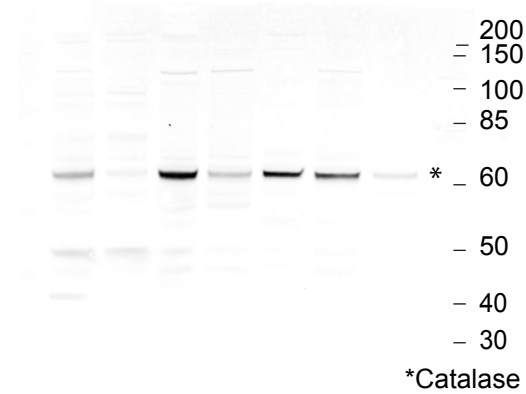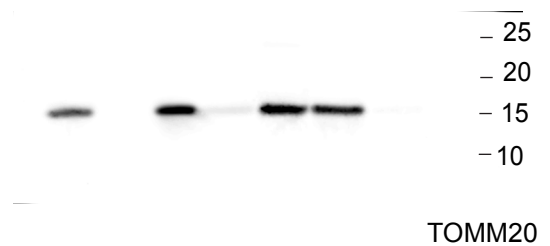

FigEV3B

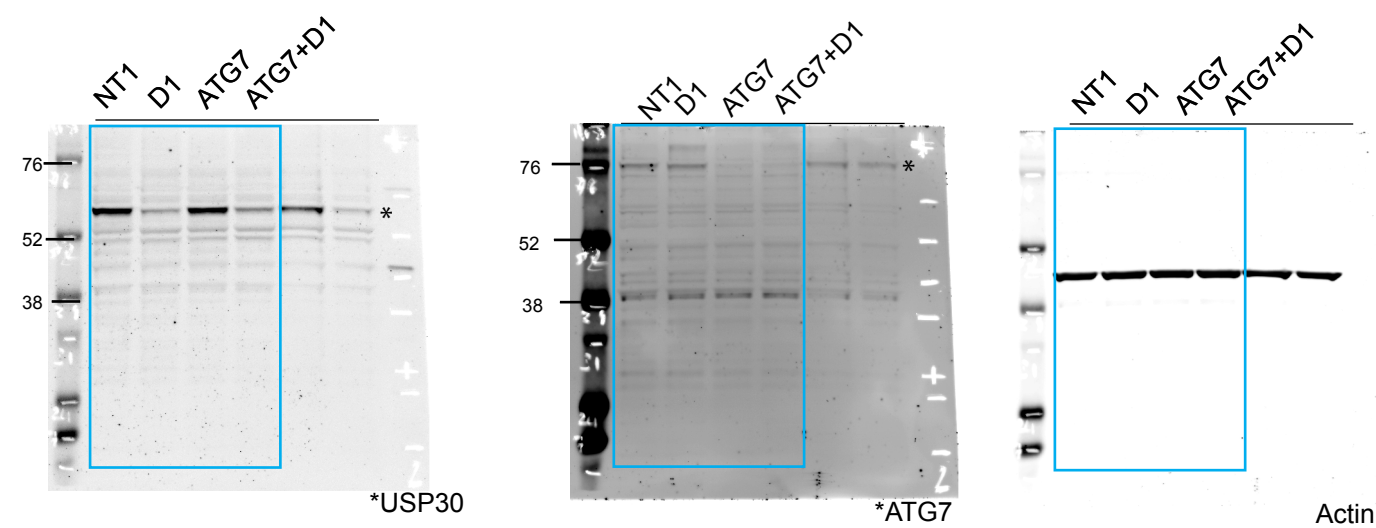

FigEV3F

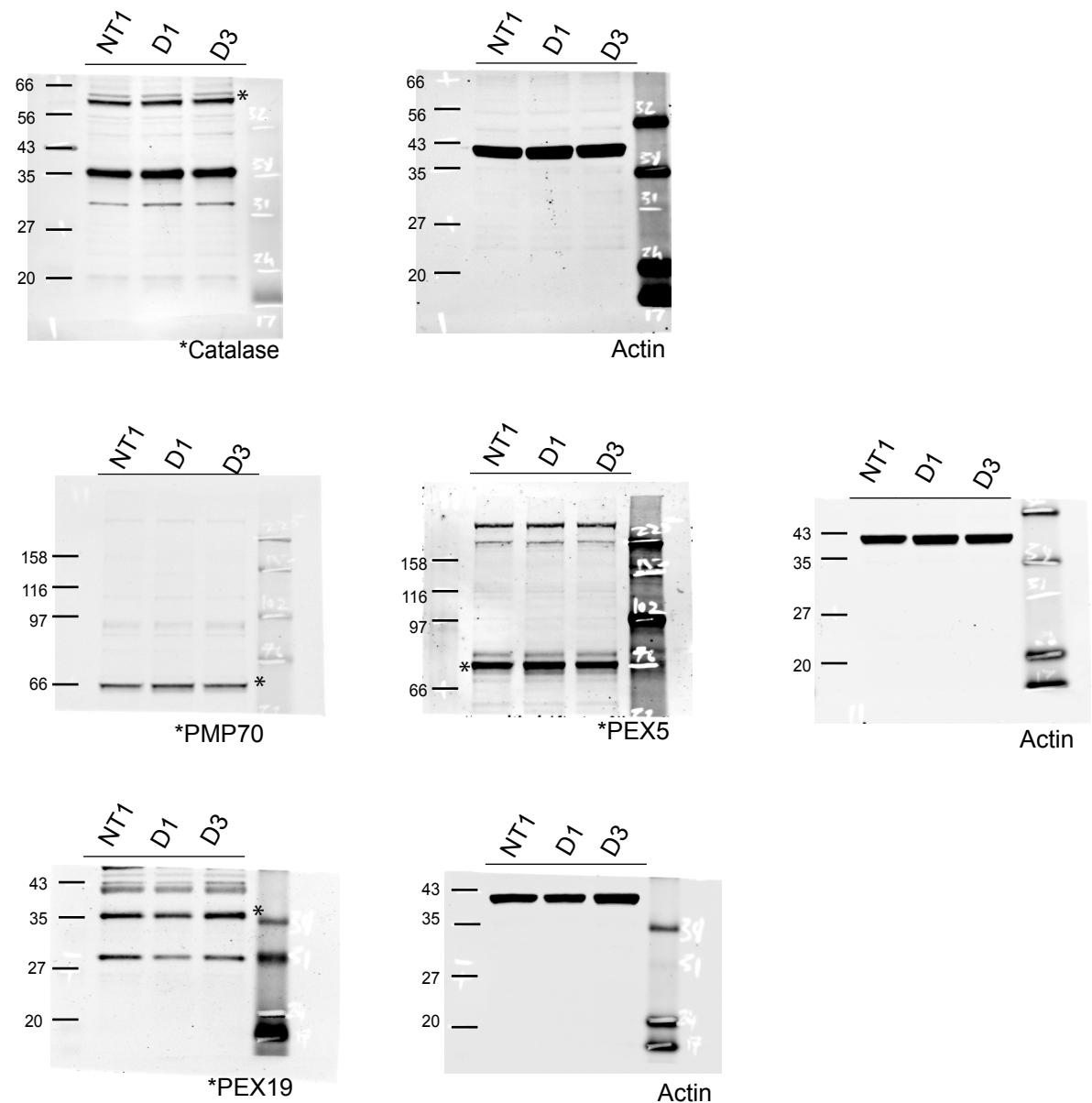

FigEV3G

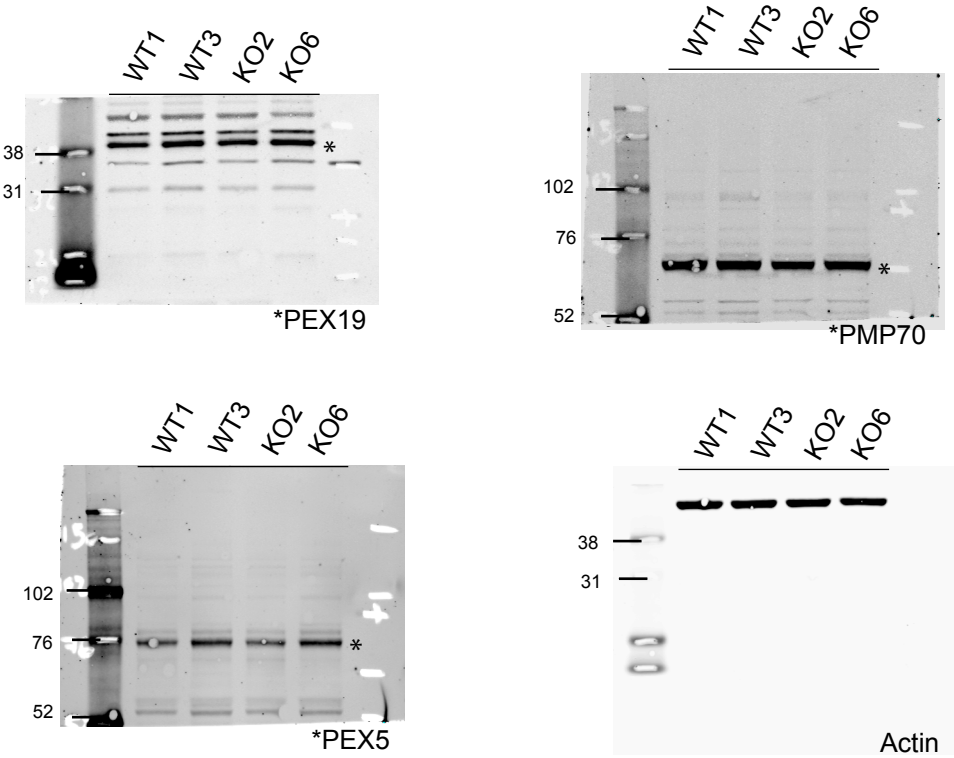

Supplement: Supplementary file 4 — Source Data for Expanded View and Appendix [file EMBR-19-e45595-s008.zip › embr201745595-sup-0008-SDataEV.pdf]

App1A

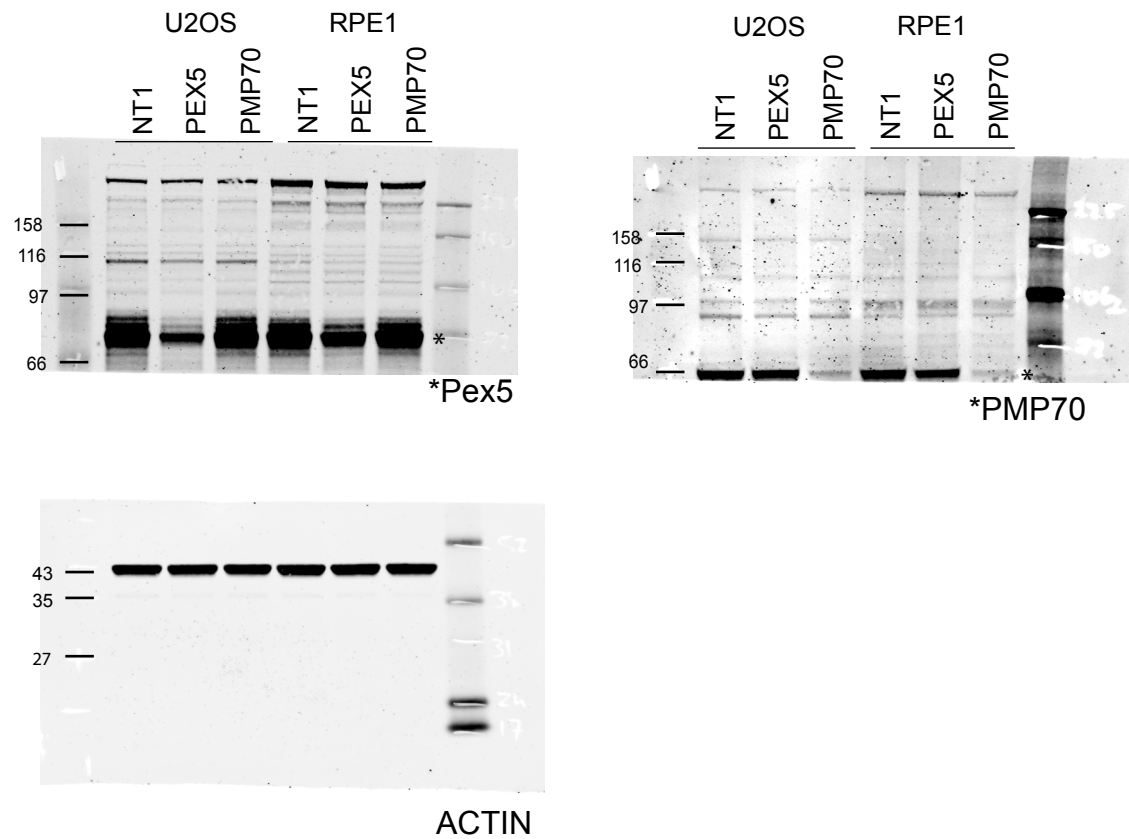

App1B

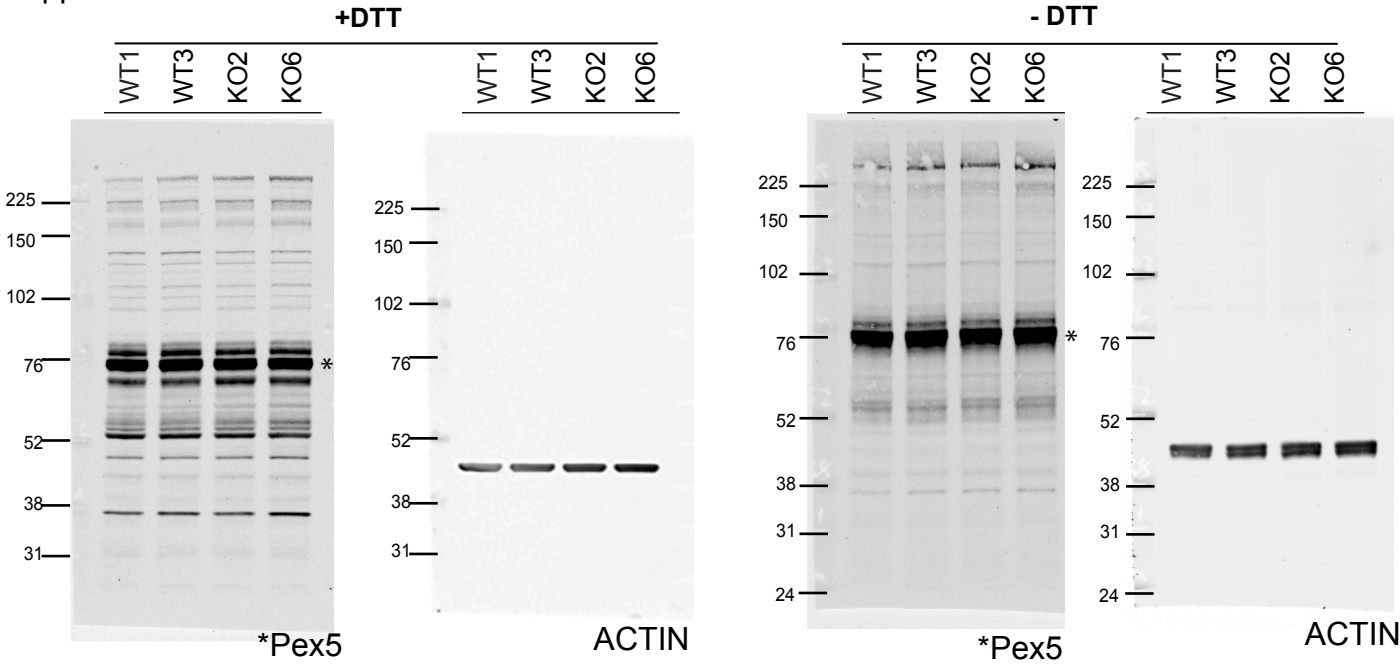

App1E

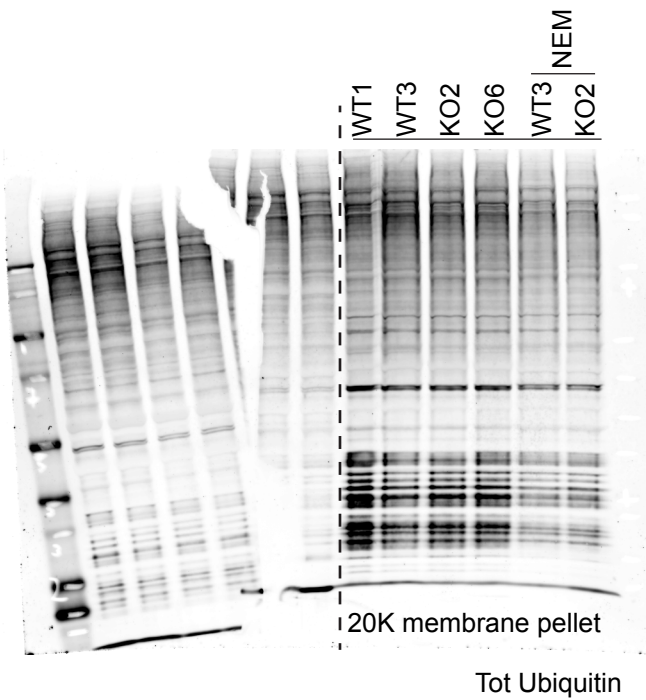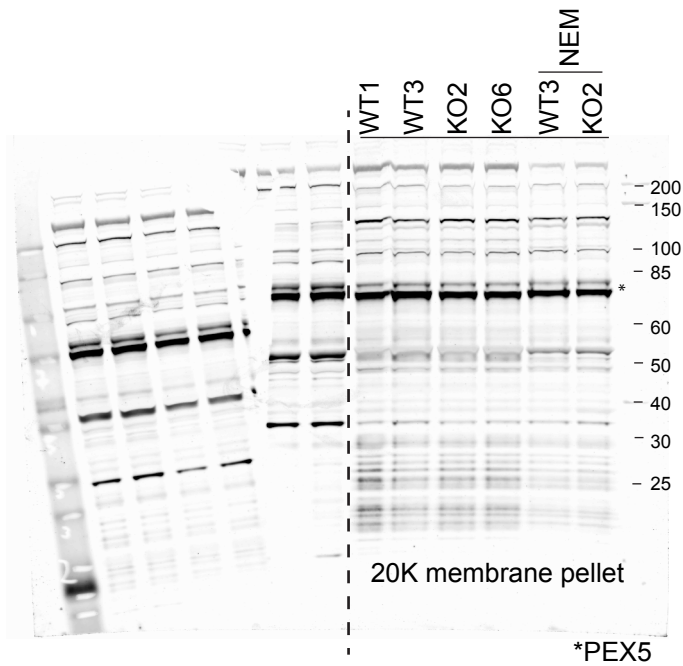

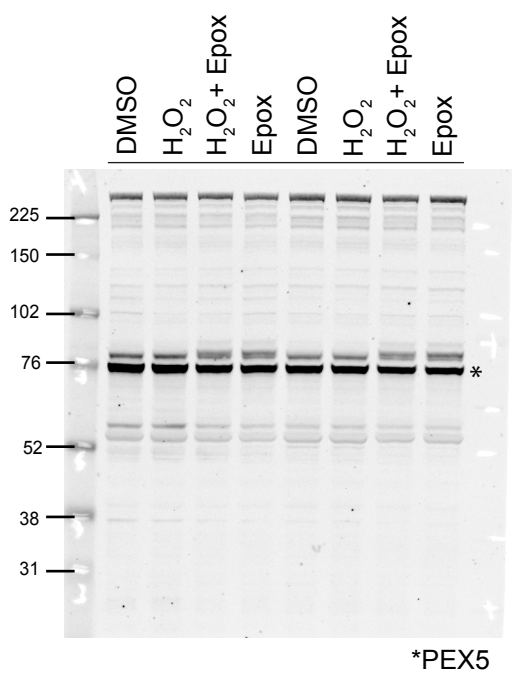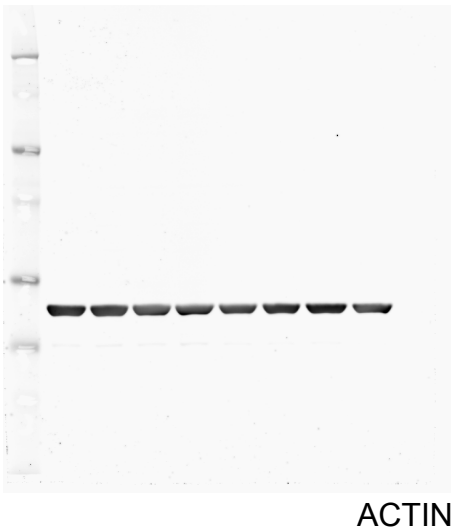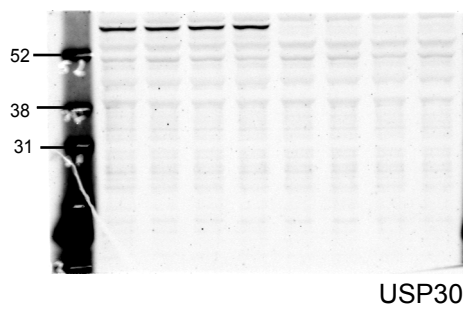

App1D

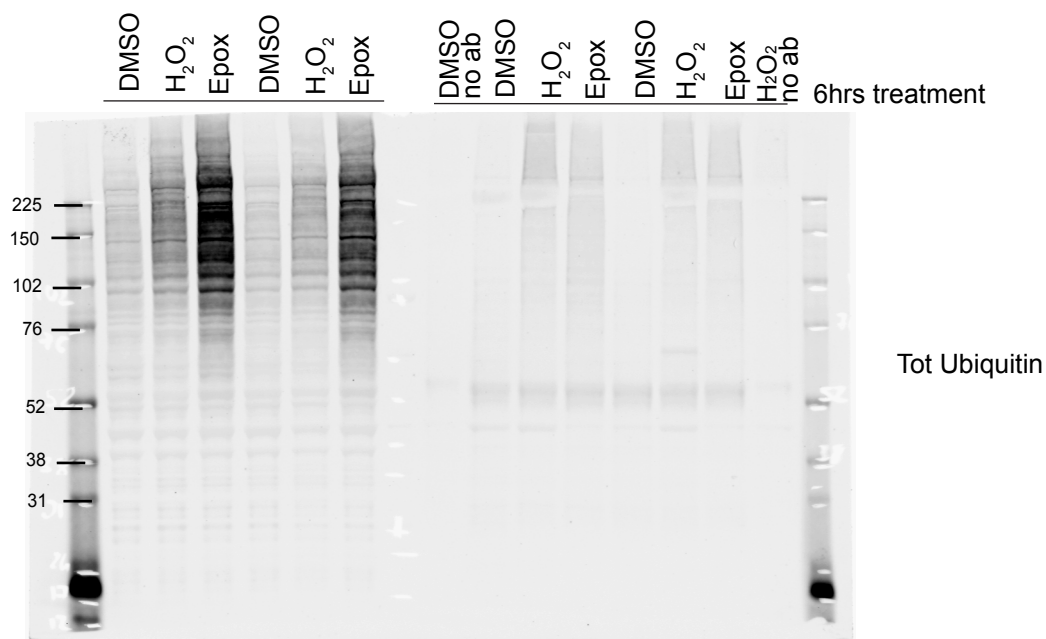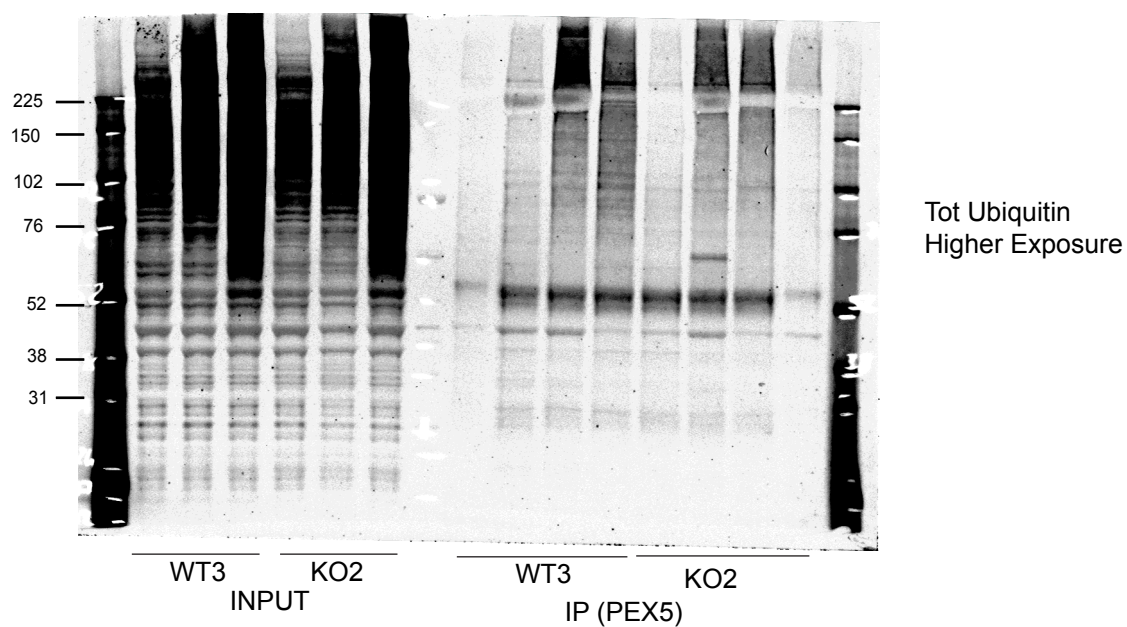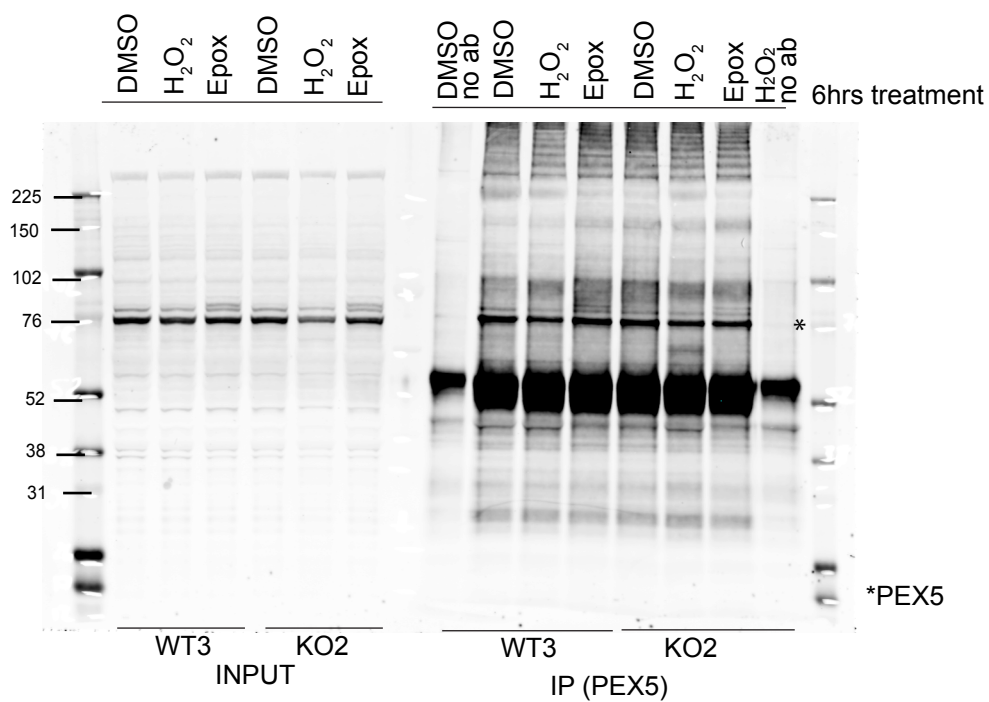

Ap2A

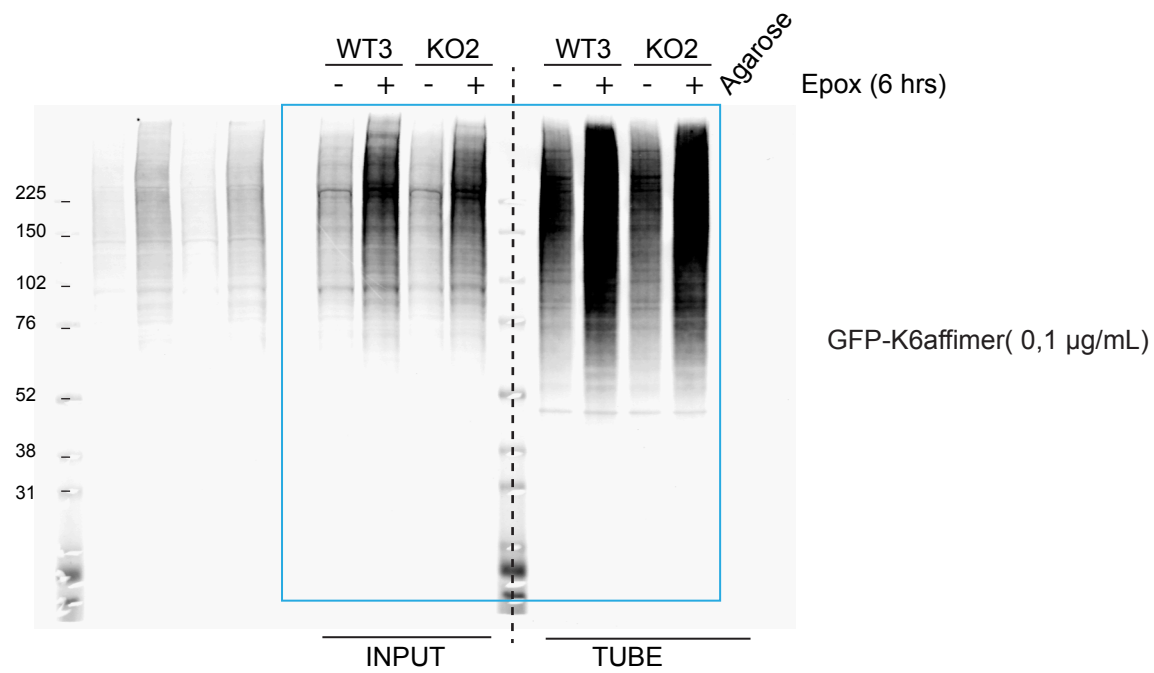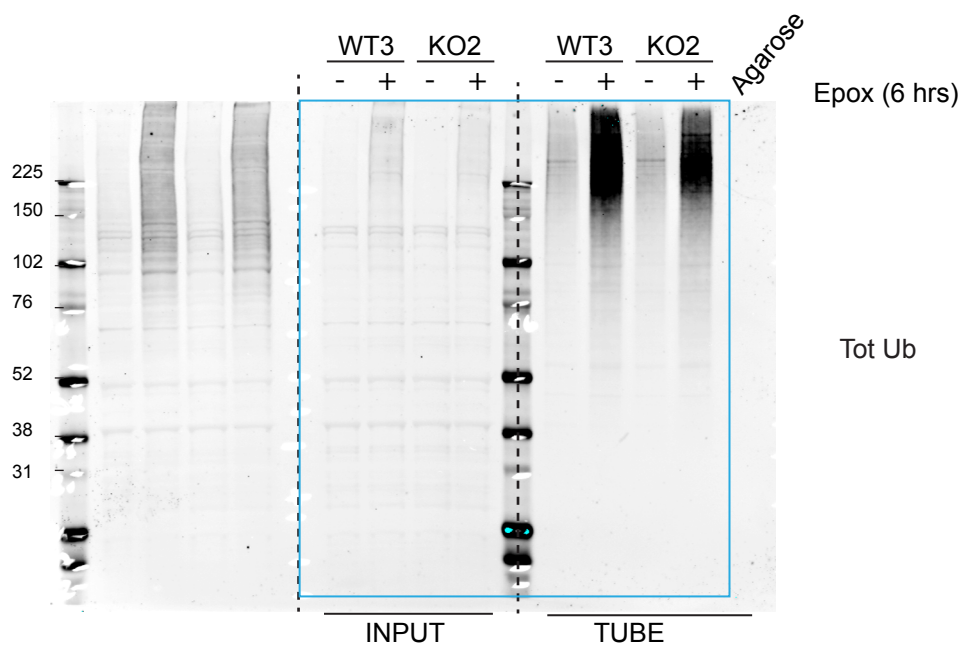

Ap2B

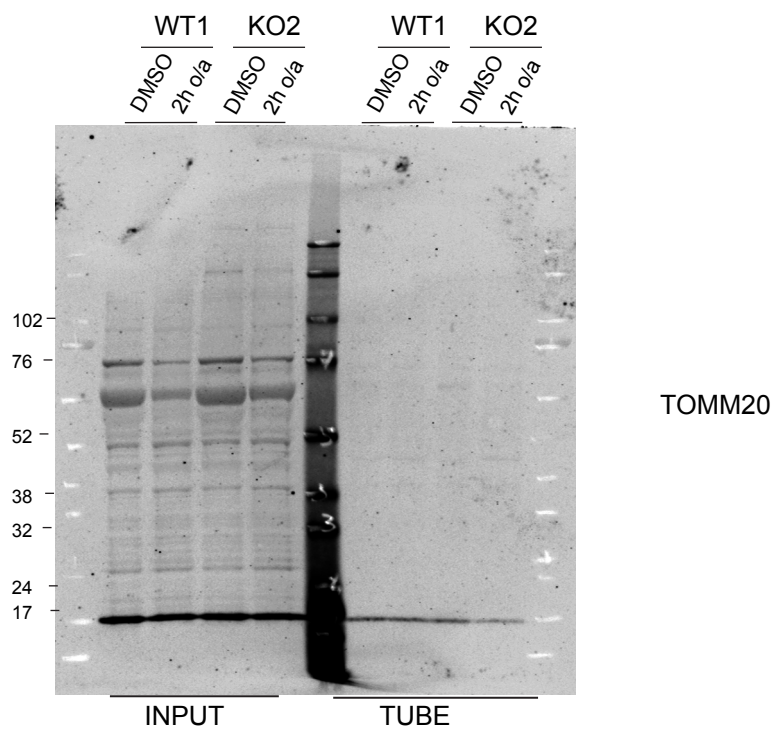

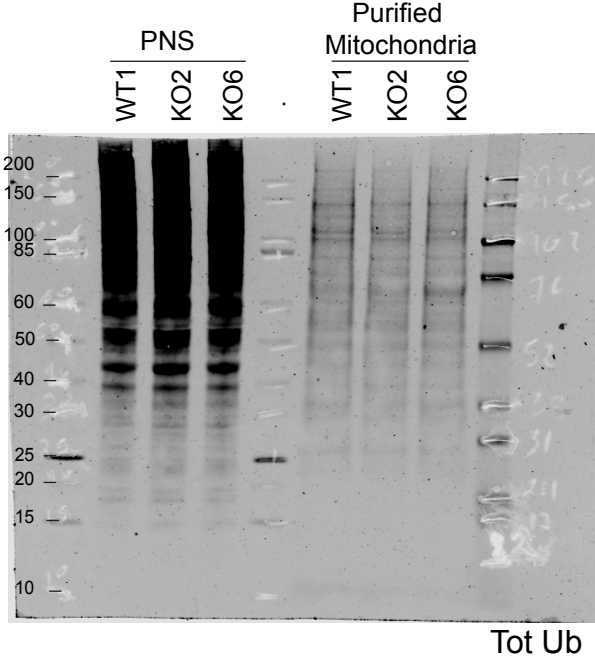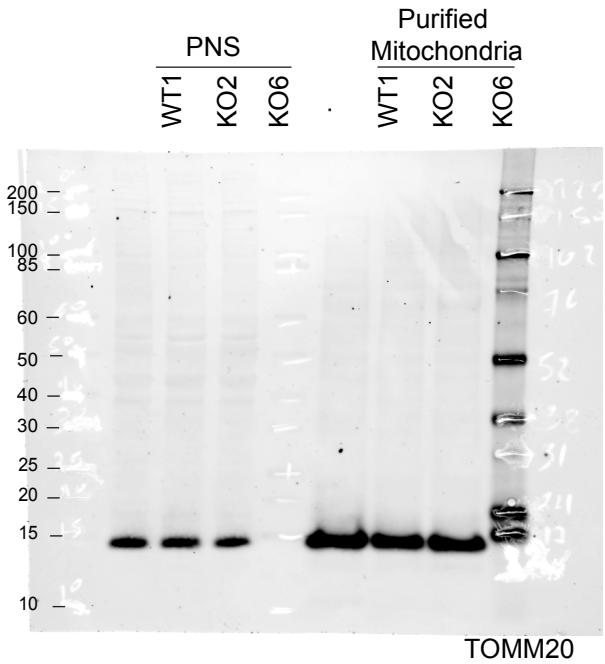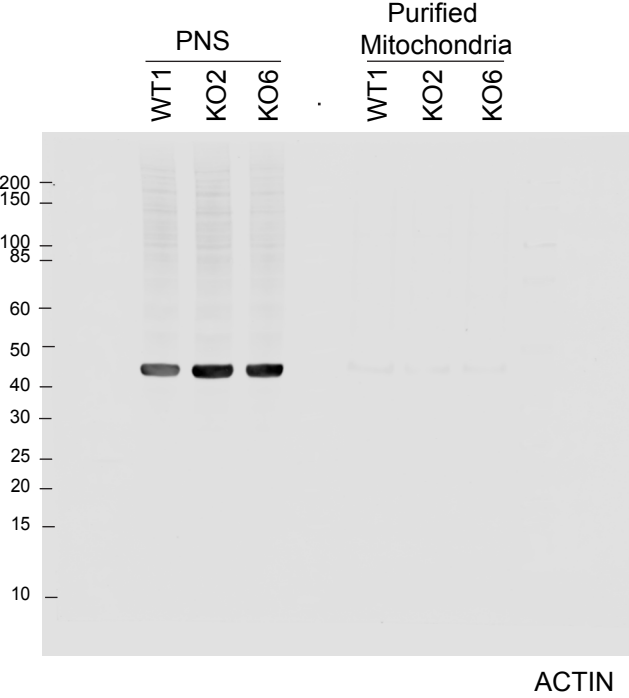

Supplement: Supplementary file 4 — Source Data for Expanded View and Appendix [file EMBR-19-e45595-s008.zip › embr201745595-sup-0009-SDataFigsS1-S2.pdf]

Fig1C

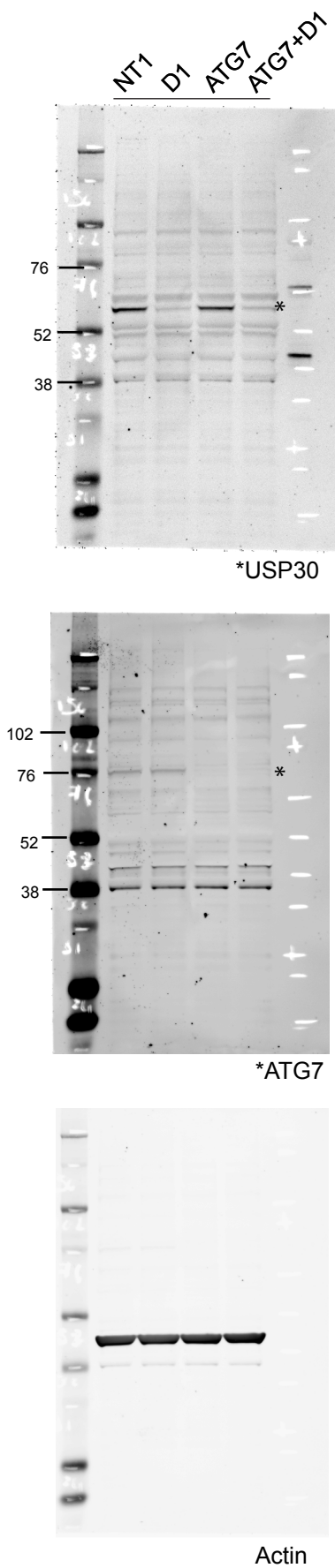

Fig1D

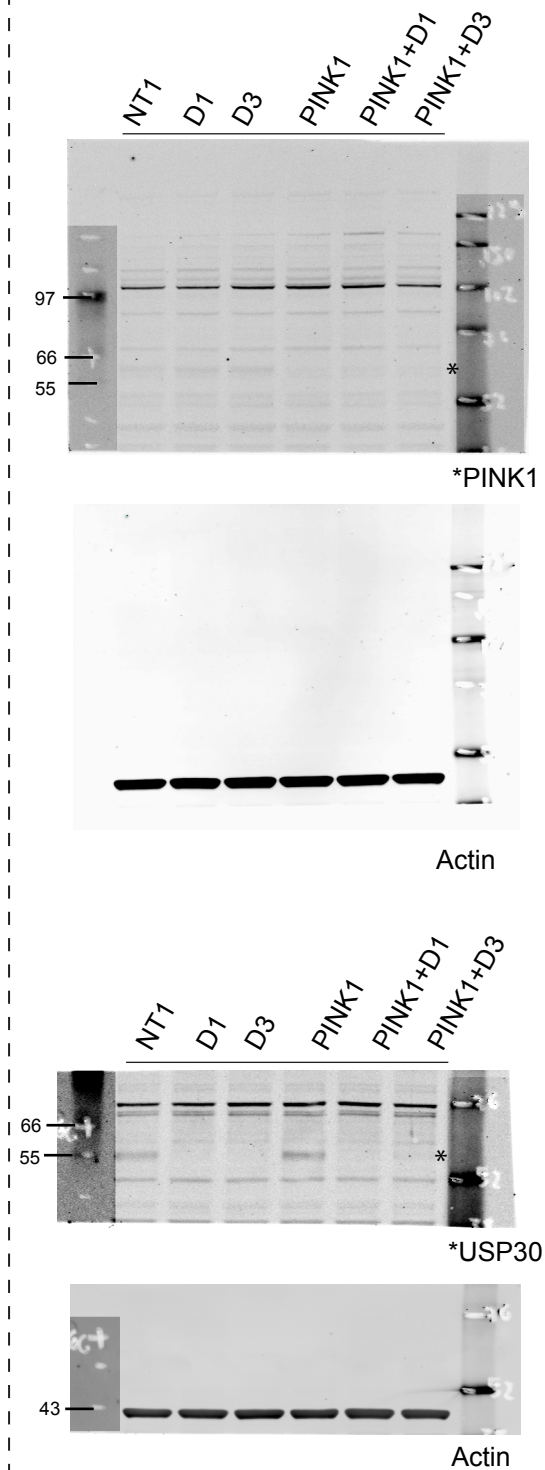

Fig1E

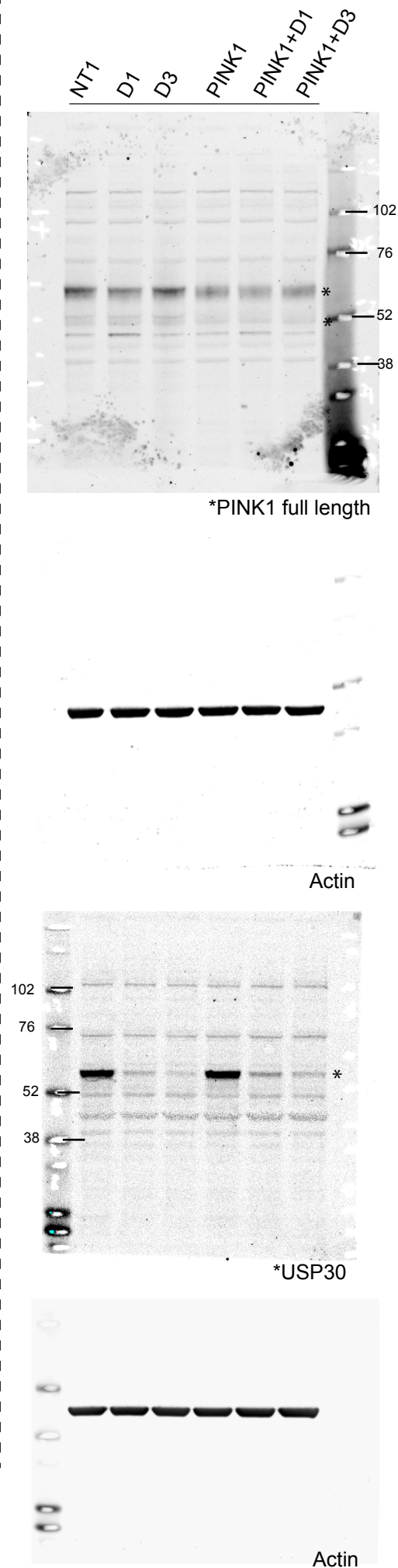

Supplement: Supplementary file 6 — Source Data for Figure 1 [file EMBR-19-e45595-s004.pdf]

Fig2C

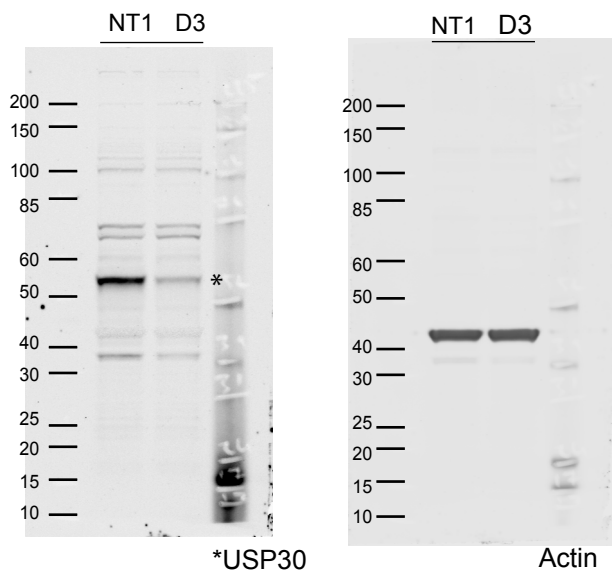

Fig2D

USP30

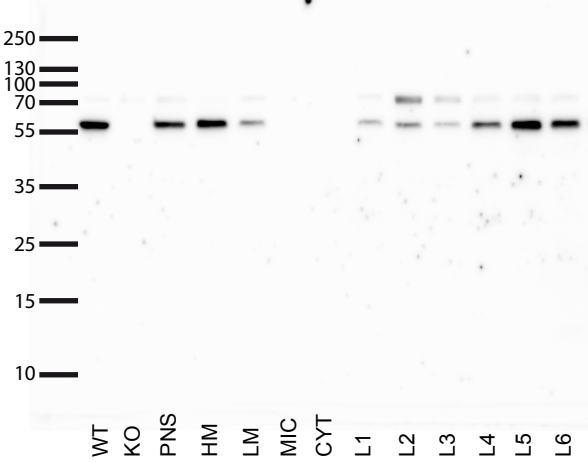

GSTK1

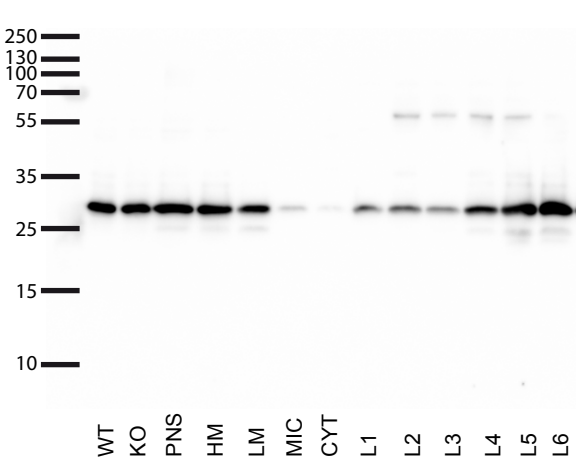

PMP70

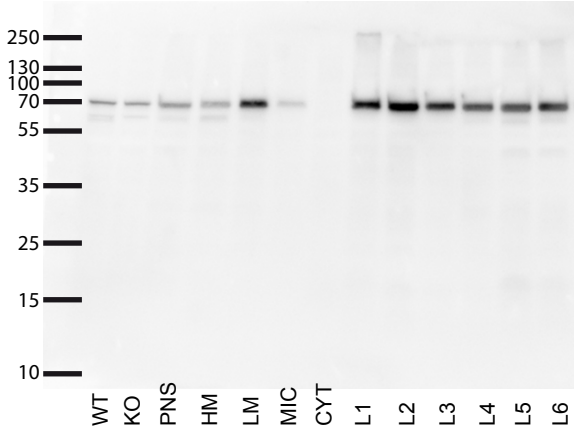

ACOX1

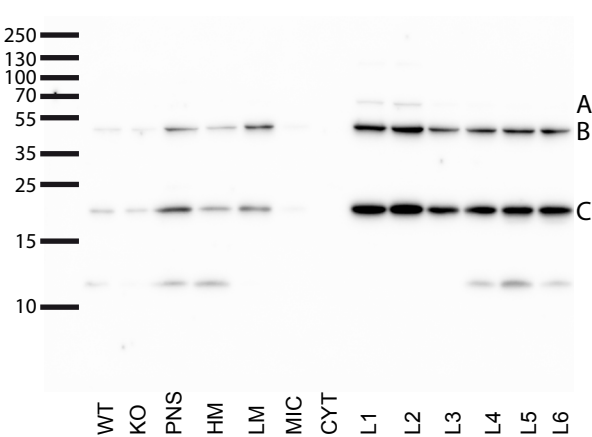

TOMM20 after PMP70

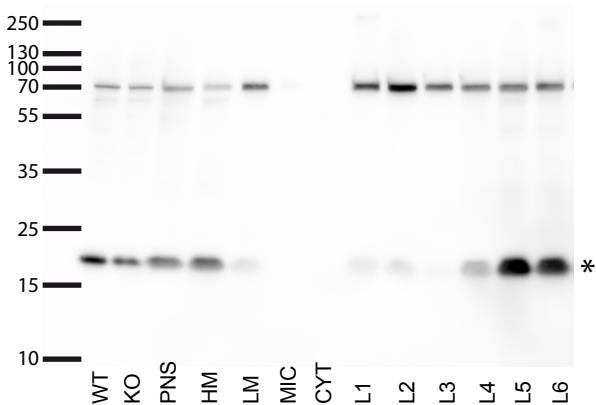

VDAC1 after ACOX1

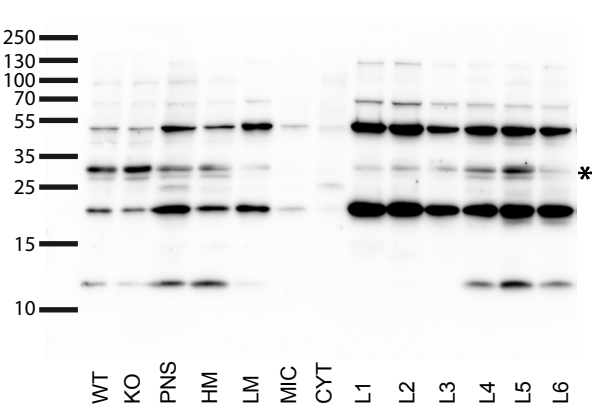

Supplement: Supplementary file 7 — Source Data for Figure 2 [file EMBR-19-e45595-s005.pdf]

Fig3A

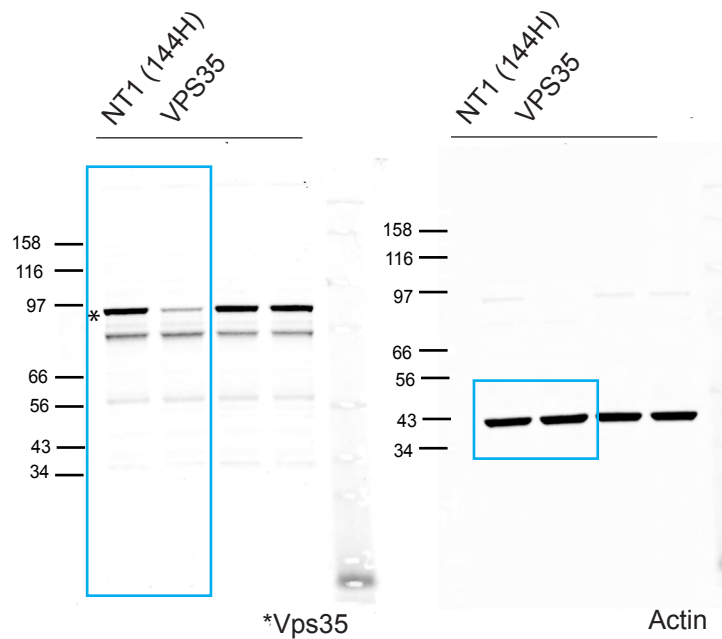

Fig3C

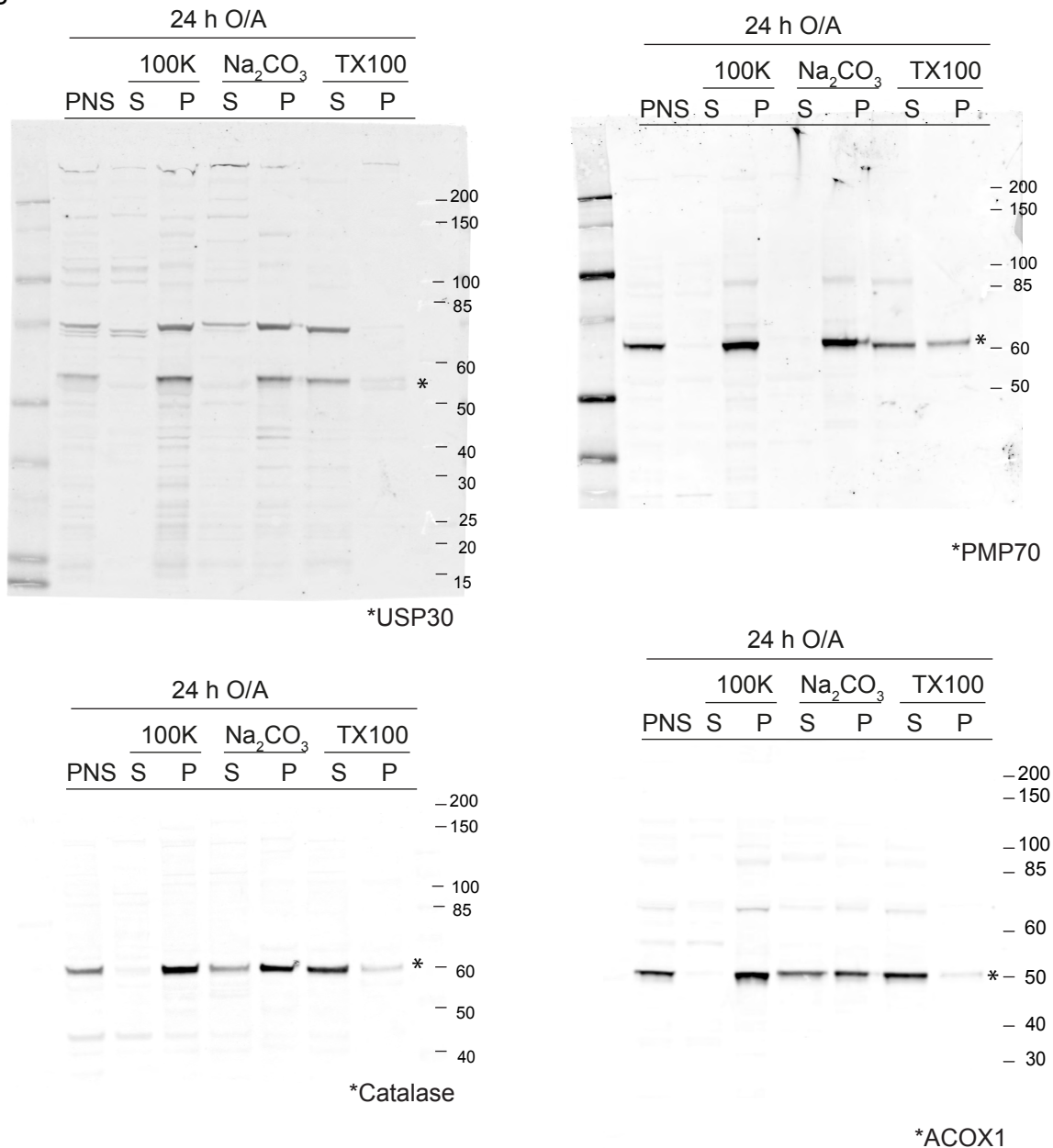

Fig3D

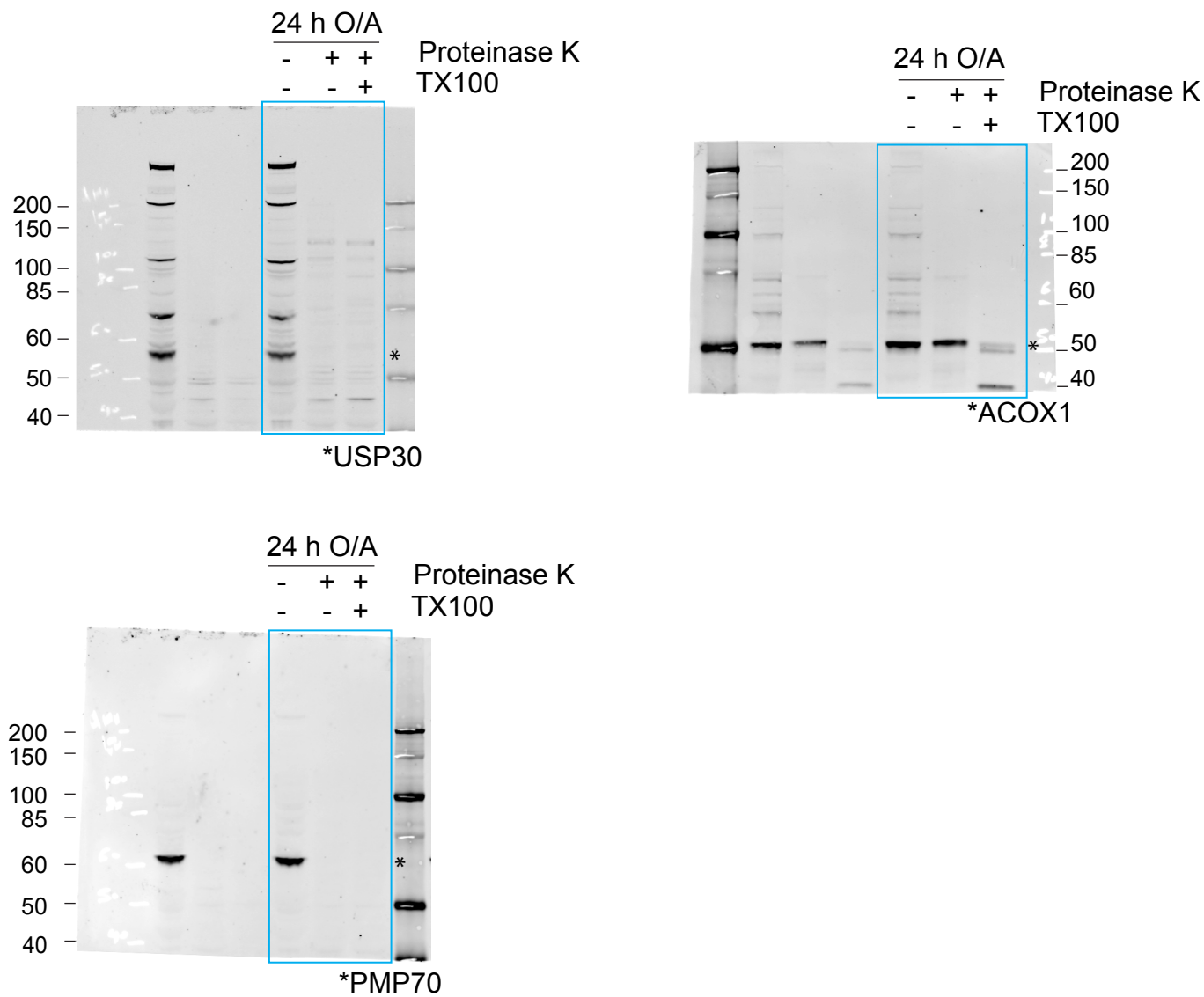

Supplement: Supplementary file 8 — Source Data for Figure 3 [file EMBR-19-e45595-s006.pdf]

Fig5A

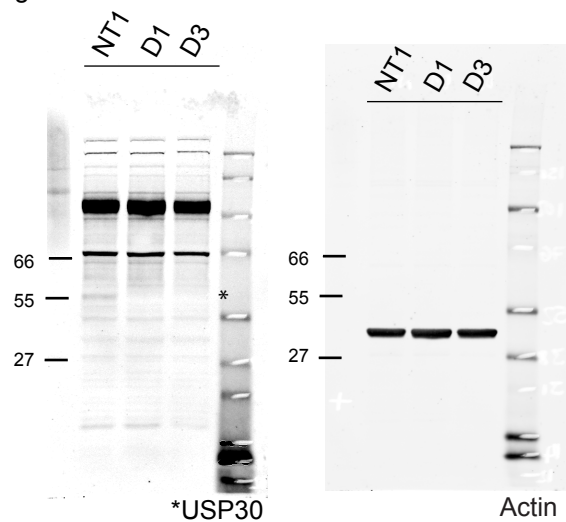

Fig5F

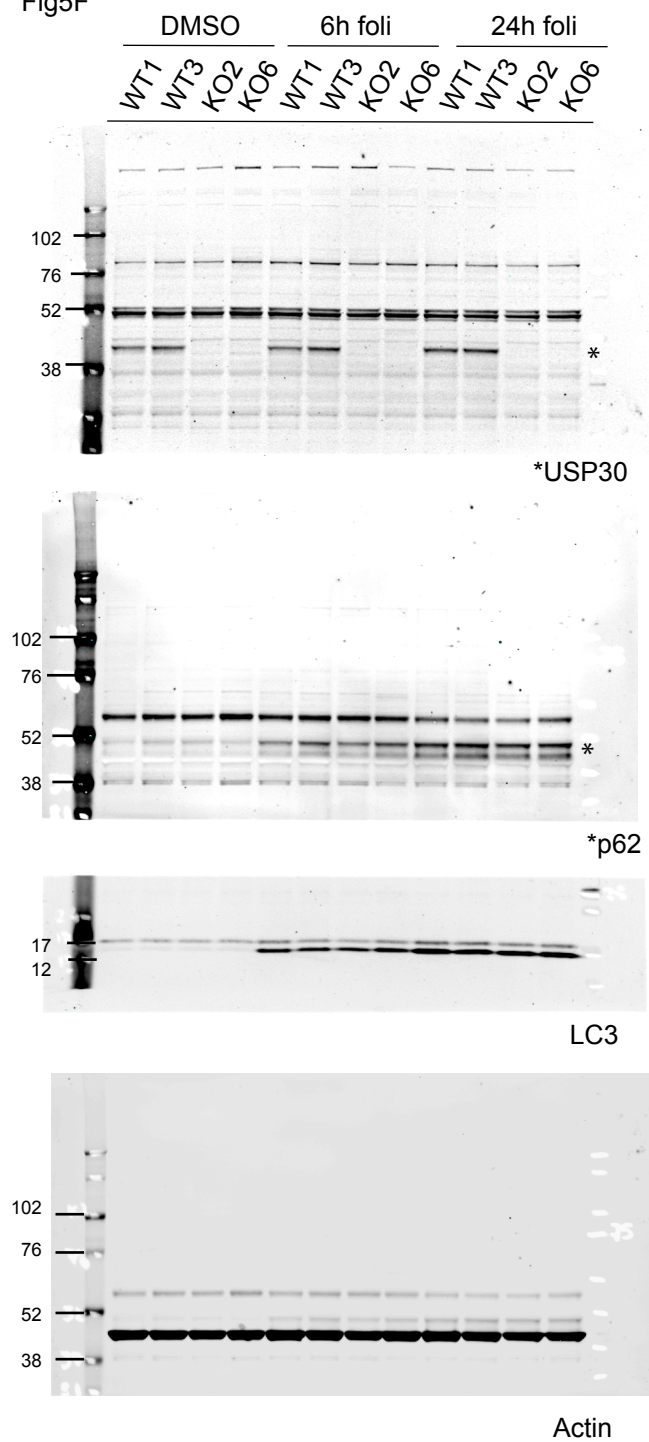

Fig5G

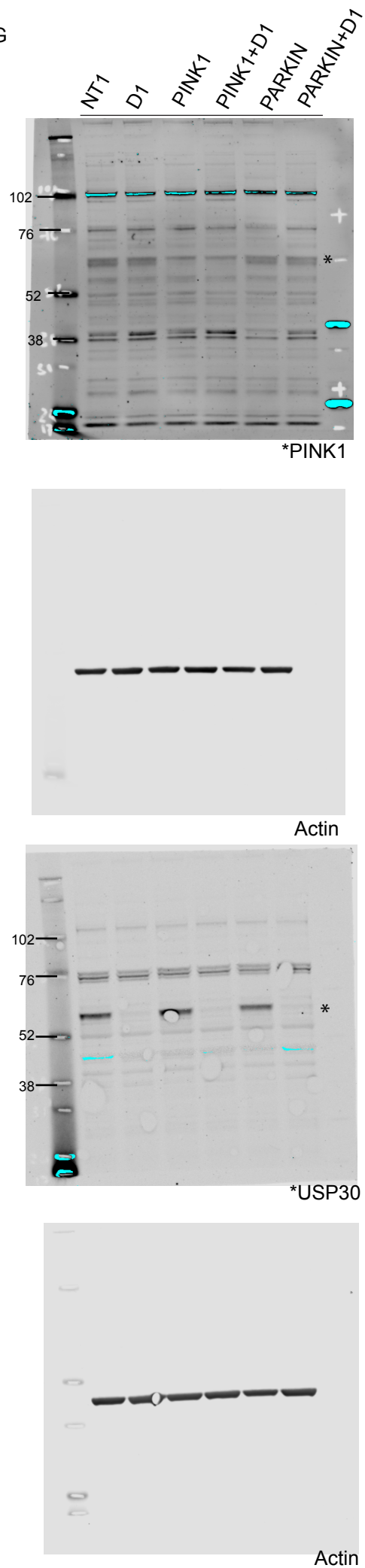

Supplement: Supplementary file 9 — Source Data for Figure 5 [file EMBR-19-e45595-s007.pdf]
